# Effect of social media overload on college students’ academic performance under the COVID-19 quarantine

**DOI:** 10.3389/fpsyg.2022.890317

**Published:** 2022-08-29

**Authors:** Yan Xu, Yilan Li, Qingfang Zhang, Xianghua Yue, Yan Ye

**Affiliations:** ^1^School of Transportation, Fujian University of Technology, Fuzhou, Fujian, China; ^2^Guangdong University of Petrochemical Technology, Maoming, China; ^3^Stamford International University, Bangkok, Thailand; ^4^Zhengzhou Technology and Business University, Zhengzhou, China; ^5^School of Economics and Management, Xiangnan University, Chenzhou, China; ^6^Graduate School of Education, Stamford International University, Bangkok, Thailand

**Keywords:** boredom proneness, information overload, communication overload, system feature overload, academic performance

## Abstract

Features and relevant services of online social media have been attracting users during the COVID-19 pandemic. Previous studies have shown that college students tend to use social media more frequently than other groups. However, in being affected by social media overload, the social media use behaviors of many college students have been out of their control in terms of their capabilities or cognition. Based on the stressor–strain–outcome (SSO) model and the theory of compensatory internet use (TCIU), we developed a research model to study the causes of social media overload and its impact on college students’ academic performance during the COVID-19 pandemic. A total of 441 valid responses from college students through questionnaires in China are collected *via* purposive sampling and used in the data analysis. This study conducts PLS-SEM to analyze collected data, finding that boredom proneness is associated with overload (stress), which has a bearing on social media overload (strain) and the reduction in final performance (outcome). Through illustrating the psychological and behavioral conditions that hinder the academic performance of students, this study provides deeper insights into students’ uncontrollable use of social media. Moreover, with respect to the identified antecedents, this study aims to find solutions to mitigate the impact of social media overload resulting from boredom proneness on the academic performance of college students.

## Introduction

The COVID-19 pandemic has dramatically hit the social operation in the world, resulting in a diversity of risks and challenges facing organizations (Cao; Said et al., 2021). In 2021, some regions of the Chinese mainland implemented lockdown policies to curb COVID-19. Boredom proneness during the lockdown period caused the improper use of social media, which may have affected the psychological health of individuals, especially those born between the mid-1990s and early 2010s (Priporas et al., 2017). Thus, we took college students under quarantine and lockdown policies as the research samples to discuss whether college students were experiencing overload triggered by boredom proneness during quarantine. Affected by the COVID-19, many university cannot provide suitable studying environment, and even the class suspension, so the overload intention may come about when the psychological health of individuals. Boredom proneness is described as “the lack of interest in and the difficulty in focusing on current activities” (Mikulas and Vodanovich, 1993; Fisher, 1998; Kass et al., 2010). Opinions given in this study contribute to deepening awareness and understanding of the excessive use of social media among college students (Bright et al., 2015; Li, 2018; Salo et al., 2018; Turel et al., 2019). Most of the existing studies explore how to enhance classroom collaboration using social media and promote the use of social media in a higher education context (Al-Rahmi et al., 2018) but neglect the correlation between boredom proneness, social media overload and the academic performance of students (Cao and Sun, 2018). The ability to develop evidence-based interventions and solutions is compromised as existing research has largely overlooked the connection between boredom, social media use, and social media overload (Cao and Sun, 2018).

The excessive use of social media may result in overload. We define social media overload according to three common dimensions: information overload, communication overload and system feature overload (Bowker, 2008). Seldom has research investigated the antecedents of information, communication, and system feature overloads on social media (Lee et al., 2016). Previous research has mainly concentrated on the negative correlation between students’ use of social media and student engagement (Junco, 2015; Datu et al., 2018). For instance, multitasking behaviors provided by social media are correlated with student engagement (Junco and Cotten, 2012; le Roux and Parry, 2017) and well-being (Becker et al., 2013). Although the wide use of social media may cause stress for students and thus affect their academic performance (Cao et al., 2018), seldom have studies used theoretical mechanisms to demonstrate how social media affects the academic performance of students. Giunchiglia et al. (2018) confirmed that the duration of usage social media apps negatively affected students’ academic performance. We aim to expand existing studies using the stressor–strain–outcome (SSO) model in combination with the theory of compensatory internet use (Davis and Luthans, 1980) and to further discuss how boredom proneness causes the perception of social media overload (organism) and how the social media overload subsequently results in a reduction in academic performance (outcome).

The daily life of college students has changed enormously due to the outbreak of COVID-19 (Khanra et al., 2021). College students are the major user group of social media, with a use level higher than average (Maier et al., 2015; Turner, 2015). The enforcement of lockdown policies on the Chinese mainland gradually led to an issue regarding the use of social media among college students, resulting in a deviation from its original intended use (Liu et al., 2021). Due to their intense usage of and limited external control over their Internet use, extensive free time, and flexible schedules, university students are more disposed than others to develop problematic social media use (Turel and Qahri-Saremi, 2016). In this paper, we investigate the etiology and consequences of social media overload among university students, the phenomenon in which the extensive adoption and use of social media has exposed people to a massive amount of information and communication demands that may require energy and cognitive processing beyond their capabilities (Lee et al., 2016). In addition, since the COVID-19 broke out in 2019, previous literature has failed to consider special context factors and cannot be used to demonstrate the reasons and psychological mechanisms for social media overload appearing in the lockdown period. Different from previous research, we first of all emphasize that the boredom proneness brought about by quarantine policies has led to social media overload among college students. Second, we investigate a situational state during the quarantine period, which has shaped a process stemming from inner feelings, which generates the social media overload and causes the decline in college students’ academic performance. We have found that social media overload results from the interaction of boredom proneness with three dimensions of overload (information overload, communication overload and system feature overload), thus ultimately affecting the academic performance of college students.

## Literature review and theory development

### Theory of compensatory internet use

The TCIU is a contemporary theory that has been widely applied in social media as an extension of the uses and gratifications theory (Elhai et al., 2017, 2020; Tandon et al., 2020). The novelty of TCIU lies in its particular focus on psychopathology as a motivator of problematic internet or social media use (Elhai et al., 2017). Information and communication overload can occur when people turn to social media in order to alleviate their boredom. Therefore, information overload and communication overload are conceptualized as strain factors. Strain can lead to various negative outcomes, such as dissatisfaction, emotional exhaustion, fatigue, or even the discontinued negative with academic performance (Ragu-Nathan et al., 2008; Tarafdar et al., 2011; Maier et al., 2015). As stated by TCIU, individuals may overuse technologies (e.g., social media) to deal with or compensate for social needs that are perceived as lacking (Wang et al., 2018) and negative emotions or stressors related to their living environment (Wolniewicz et al., 2018). On the basis of TCIU, we consider that college students who are experiencing boredom proneness, which is a negative emotion (Tandon et al., 2021), would increase their use of social media to cope with and compensate for it. We hold the same arguments as those of previous studies that individuals are more inclined to deal with perceived negative emotions (Tandon et al., 2020) and unsatisfied requirements (Wang et al., 2018; Wolniewicz et al., 2018) through social technologies, such as smart phones and social media. Thus, we consider TCIU an appropriate theory to establish the theoretical framework of our study. However, some scholars assert that TCIU has been focusing on the psychopathology and negative emotions, making it inadequate for providing theoretical support for social media use behaviors (Wolniewicz et al., 2018). As a result, we contend that TCIU is not able to offer a comprehensive outlook for the study of the proposed associations, especially overload. Therefore, we complement TCIU with the SSO model to consider the associations between boredom proneness, overload and fatigue. In this paper, we investigate the etiology and consequences of social media overload among university students, the phenomenon in which the extensive adoption and use of social media has exposed people to a massive amount of information and communication demands that may require energy and cognitive processing beyond their capabilities (Lee et al., 2016).

### SSO model

Our research model has been developed based on TCIU to explain the stress process combined with the SSO model (Koeske and Koeske, 1993). The SSO model developed by Koeske and Koeske (1993) was originally applied in psychology studies to describe the stress process. On the grounds of this framework, stressors exert an influence on users, thus leading to their behavioral outcome. In the SSO model, stressors are defined as environmental stimuli that are, as believed by individuals, stimulating, troublesome or destructive. Common stressors include overload, conflict and intrusion (Ayyagari et al., 2011; Bucher et al., 2013; Maier et al., 2015). Strain is an individual’s psychological response to stressors and may have a frustrating effect on an individual’s attention, physical condition and emotion (Cheung and Tang, 2010). Studies of strains, such as emotional exhaustion and overload, are very widely conducted in relevant studies (Choi et al., 2014; Zhang et al., 2016). In organizations, stressors are the conditions for factors generating stress, and strain is the psychological outcome of stress. Furthermore, stress is a modulator for the effect of perceived stressors on outcome variables (Lee et al., 2016). Prior studies of stress contend that reduced organizational commitment, poor job performance and discontinuity of intention are the outcomes of stress (Ragu-Nathan et al., 2008; Kim et al., 2012). In the past, the SSO model was commonly used to study a stress-related situation and its outcomes in the context of technology use (Ragu-Nathan et al., 2008; Ayyagari et al., 2011; Dhir et al., 2018). For example, Cheung and Tang (2010) investigated how job stressors (including job characteristics and emotional dissonance) affect employees’ subjective health and work outcome (Choi et al., 2014). Studies have shown that social stress related to customers has a positive effect on emotional exhaustion, which, in turn, has a negative effect on customer orientation and the performance of service restoration. These studies mainly discuss job stress in workplaces. As social media has been extensively used among college students, they are more likely to spend a lot of time on social media, resulting in the excessive use of social media related to technology stress and further bringing about negative outcomes (Pavlou et al., 2007; Aladwani and Almarzouq, 2016). In the context of COVID-19, the excessive use of social media and the social media overload caused by boredom proneness among college students constitute stressors that affect individuals’ emotions and attitudes (e.g., overload, regret, or discontent) toward social media, and these stressors will further give rise to adverse outcomes such as a decline in academic performance (Cao and Sun, 2018; Dhir et al., 2018; Nawaz et al., 2018; Yu et al., 2018). This is entirely in line with the major purpose of this study, that is, how stress-related factors trigger social media overload and thus a decline in academic performance (outcome). Consequently, we use the SSO model as the theoretical basis to discuss the negative effect of social media overload resulting from boredom proneness on the academic performance of college students.

### Boredom proneness and overload

To be sure, social media is able to facilitate friendship development and maintenance, social contact well-being and knowledge exchange among college students, but it may bring about negative outcomes when it is used beyond individuals’ available resources and energy (Karr-Wisniewski and Lu, 2010). In the context of social media, overload is the key factor for such negative outcomes (Lee et al., 2016). The overload derives from the imbalance between the unexpected demands in the context and the limited handling ability of individuals (Edwards and Cooper, 1990).

Overload, as a typical stressor, has been extensively examined in different research fields such as work overload (Ayyagari et al., 2011), connection overload (LaRose et al., 2014) and social overload (Maier et al., 2012), for some people, this has resulted in overload, which refers to a mismatch between environmental requirements and an individual’s ability to cope. The topic of overload has been studied by many scholars, who argue that overload can be divided into information overload, communication overload and system feature overload (Bowker, 2008). Information overload will appear when the information on social media is too much to be handled properly by an individual, especially when the information is presented in an excessively fast and discontinuous manner. Users reported feeling low on energy and unable to concentrate on important tasks after a period of heavy social media use (Whelan et al., 2013). Communication overload refers to when individuals’ communication skills are challenged when too many communication demands are embedded in social media. System feature overload refers to when the features provided on social media exceed the users’ demands (Thompson et al., 2005). To be specific, the younger generations, like college students, are the major participants in social activities on social media (Kirschner and Karpinski, 2010), and have a strong desire for social contact. Since the interpersonal relationship in a social network is always reciprocal, individuals feel that they are obliged to meet the demands of online friends in the form of emotional or material support (Maier et al., 2012). Individuals are likely to become exhausted in the face of an increasing number of social support requests if they are not able to satisfy such requests, and they may also feel uncomfortable regarding their too frequent responses to such social requests, thus leading to a perception of social contact burden (Maier et al., 2015). Once the number of online social requests goes beyond the handling ability of users, a troublesome environmental stimulus will be generated, leading to a series of emotional and behavioral reactions (Evans et al., 2000). This unwholesome state has been proven by experience to be a stressor that exerts a negative influence on one’s personal life (Maier et al., 2015). Similar to the way that boredom proneness has been found to generate stress in social media users (Whelan et al., 2013), we conceptualize boredom proneness as a stressor for the same population. For example, learners must pay attention to learning materials and other activities, such as familiarizing themselves with increasingly complex SNS system features, responding to demanding social support from friends, confronting the distractive advertising, etc. These overloads lead to negative emotional responses such as stress, frustration, and anxiety, which also occur in learning communities. When individuals feel threatened by the physical and psychological strain generated by SNSs, they have to prevent or reduce the adverse outcomes and restore emotional stability, thus threatening the sustainability of SNS’s massive benefits for people. Therefore, we consider information, communication and system feature overloads as stressors and three dimensions of overload related to the overuse of social media.

College students might have sought out other experiences to relieve boredom proneness during the quarantine and lockdown period, even though these experiences might have caused negative impact (Bench and Lench, 2013). Boredom proneness is an undesirable feeling, so the human brain looks for external stimuli to avoid it. Social media provides almost endless experiences, which attract scores of people to pass time and seek out spiritual and emotional stimuli. However, excessive stimuli may be a bad thing. Prior studies of addiction have mentioned that boredom proneness can result in some adverse outcomes, such as social media overload. Social media become a kind of pathological pursuit for users to relieve their boredom proneness, thus resulting in the issue of overload (Turel et al., 2014, 2016, 2019). Many prior studies have explained why people overuse social media, and boredom proneness is a significant influencing factor recognized by scholars; seeking out entertainment and killing time are powerful predictive factors for the use of social media platforms (Quan-Haase and Young, 2010; Ku et al., 2013). Boredom proneness drives people to seek more stimulating activities in social media (Bench and Lench, 2013; Alter, 2017), which then lead to information, communication and system feature overloads.

According to the logic of addiction studies described above, adverse outcomes will bring about information, communication and system feature overloads when people try to relieve their boredom proneness using social media. Thus, we propose the following hypotheses:

*H1*: Boredom proneness is positively correlated with information overload.

*H2*: Boredom proneness is positively correlated with communication overload.

*H3*: Boredom proneness is positively correlated with system feature overload.

### Antecedents of overload

We consider the overload examined in this study as psychological stress, which is an individual psychological state, and the behavioral outcomes that can be affected by cognitive bias (Brooks and Califf, 2017; Mahmud et al., 2017; Steelman and Soror, 2017). In previous studies, overload has been extensively investigated as a special type of psychological tension caused by overloads (Ahuja et al., 2007; Maier et al., 2015). In the social media context, an excessive amount of information and interaction may weaken user activation and make them feel tired (Maier et al., 2015). Exhaustion refers to a state of extreme overload caused by the long-term and excessive consumption of spiritual resources (Schaufeli, 1995; Ravindran et al., 2014). Moore (2000) investigate social networking service (SNS) exhaustion, which is a psychological reaction to stressors, as an outcome of social overload in the social networking environment. The investigation showed that workload is the strongest predictive factor of employee overload. In our context, overload refers to the disgust and potentially harmful and unconscious psychological reactions to stress conditions when using social media (Maier et al., 2015). It represents the tiredness arising from social media use.

Information overload will occur if the information accessible to users on social media goes beyond their ability to handle it (Zhang et al., 2016). Specifically, the information overload would have resulted if college students made responses that went beyond their management and handling abilities in order to eliminate boredom proneness during the quarantine and lockdown period. It can produce stress and negative emotions, so people cannot easily cope with requests from social media platforms, which is undesirable (Cao and Sun, 2018). Plenty of information is generated on social media as the number of users and their level of activation increases (Lee et al., 2016). As a result, college students need to be able to cope with the incessant information generated on social media, thus causing overload. The overabundance of information on social media may exceed the expected cognitive range of the human brain, making users feel overwhelmed and tired (Lee et al., 2016). The massive amount of information on social media can quickly go beyond the cognitive range of humans, making people feel at a loss (Wurman, 1990). Previous studies have shown that information overload is one of the major antecedents of social media overload in the social media context (Lee et al., 2016; Zhang et al., 2016). Confronted with the massive amount of information, users may feel out of control (Zhang et al., 2016) and may regret using social media (Cao and Sun, 2018; Nawaz et al., 2018), thereby leading to social media overload (Cao and Sun, 2018). Therefore, users may feel extremely tired on social media when they perceive information overload (Gao et al., 2018). The overload will be evident when users feel that it is difficult to manage large amounts of information and communication from others (Lee et al., 2016). Based on the above statements, we posit the follow hypothesis:

*H4*: Information overload is positively correlated with social media overload.

Social media provides a diversity of features to facilitate communication (Garrett and Danziger, 2007; Ou and Davison, 2011). Message and conversation requests from other users attracts the attention of users and excessively interferes with their behaviors, resulting in communication overload (Karr-Wisniewski and Lu, 2010; Cao and Sun, 2018). As reported by Lee et al. (2016), communication overload is a source of overload in the context of social media use. Users will perceive a communication overload if there are too many social requests to cope with and be satisfied on social media (Karr-Wisniewski and Lu, 2010). After coping with the communication requests, individuals need take several minutes to recover the interrupted work or learning (Karr-Wisniewski and Lu, 2010; Ou and Davison, 2011). Communication overload may repeatedly interrupt the daily learning tasks of college students (McFarlane and Latorella, 2002; Cho et al., 2011), making them feel tired and giving rise to more severe spiritual or physical diseases (Deutsch, 1961; Klapp, 1986). We, therefore, contend that college students often feel overload due to communication overload.

We develop the following hypothesis:

*H5*: Communication overload has a positive impact on social media overload.

System feature overload will appear if the features of applications are not suitable for current tasks or the system features are too complex to complete tasks (Thompson et al., 2005; Karr-Wisniewski and Lu, 2010; Zhang et al., 2016). In the social media context, system feature overload refers to social medial users’ perception of technology features. It can also be defined as a perception that features provided by social media exceed user demand (Thompson et al., 2005; Zhang et al., 2016). If system features on social media often change and are too complex for users, system feature overload will appear and result in adverse outcomes, such as social media overload. For example, Facebook updates its system features and user interface almost once a week (Fu et al., 2020), so users need to spend more time and attention to adapt to these frequent changes. System feature overload is particularly obvious when the design changes do not match user habits (Ayyagari et al., 2011), especially when users have become accustomed to an appearance and find the system changes overwhelming. Users may also experience system feature overload, thus leading to the perception of social media overload (Lee et al., 2016). Frequent unnecessary updates for feature interfaces will lead to social media overload (Ayyagari et al., 2011; Ravindran et al., 2014). Hence, we contend that college students may be tired of social media use and feel overloaded when they realize that the cost required to learn and use system features on social media outweighs their benefits.

Based on the above statements, we make the following hypothesis:

*H6*: System feature overload has a positive impact on social media overload.

### Relationship between overload and academic performance

Indeed, the most popular social media applications are designed to encourage compulsive use (Alter, 2017). Many people are unable to override their impulsive habitual use of social media (Turel and Qahri-Saremi, 2016) and smartphones (Soror et al., 2015). Social media users often do not get all the functionality they want from one platform and need to constantly switch between too many different (non-) technological alternatives, resulting in negative perceptions which Maier et al. (2015). The relationship between social media use and academic performance has been a hot topic in the education field in recent years (Liu et al., 2017). Meta-analysis has shown that the excessive use of social networking services has a significantly negative association with academic performance (Aladwani and Almarzouq, 2016). It has also found that the compulsive use of social media leads to problematic learning outcomes. As a result, we consider the academic performance of students as an outcome in this study.

Overload is a complex physical and psychological state. Physical overload is clinically defined as “not [being] able to maintain the required or expected strength” (González-Izal et al., 2012). Psychological overload is described as a state of overload that is related to stress and other intensive emotional experience (Shen et al., 2006). Overload is a kind of subjective inner feeling that varies from person to person (Nail and Winningham, 1995). Physical overload is more likely to appear in the mandatory environment related to physical labor but not in the context of social media use, so social media overload can be regarded as a form of psychological overload (Zhang et al., 2016). Overload brings about a variety of outcomes that decrease students’ academic performance (Junco, 2015). Academic performance means the extent to which students achieve their short-term or long-term educational goals. Prior studies have discussed all sorts of negative outcomes caused by social media use (Tarafdar et al., 2015), and reduced academic performance is the most common one. Past studies seldom demonstrate that social media overload can lead to reduced academic performance among college students (Ayyagari et al., 2011).

We contend that social media overload derives from individuals’ dependence on social media, particularly from college students’ excessive use of social media to eliminate boredom proneness during the COVID-19 lockdowns. This has been identified in recent research findings. For example, Islam et al. (2020) found that the increased use of social medial to explore new content (e.g., new information) during lockdowns may have brought about overload and have had a negative impact on academic performance. In the academic activities of college students, the focus on social media platforms may cause an explosion of psychological stress and tiredness, thus weakening their actual performance (Ayyagari et al., 2011). Psychological efforts are required for a good performance in any task (Boksem and Tops, 2008). The performance will decline when people feel tired (Van der Linden and Eling, 2006). Social media overload may have a significantly negative impact on student attention during learning (Caplan and High, 2006). As an example, other studies on American college students have stated that social media overload weakens actual academic performance (Rosen et al., 2013a,b; Junco, 2015). As reported, teachers commonly worry that social media overload will have a negative impact on the performance of students in various academic activities. Thus, we infer that social media overload has a negative effect on the academic performance of students because it distracts students’ attention from learning.

Based on the above statements, we posit the following hypothesis:

*H7*: Social media overload has a negative impact on academic performance.

**Figure 1:**
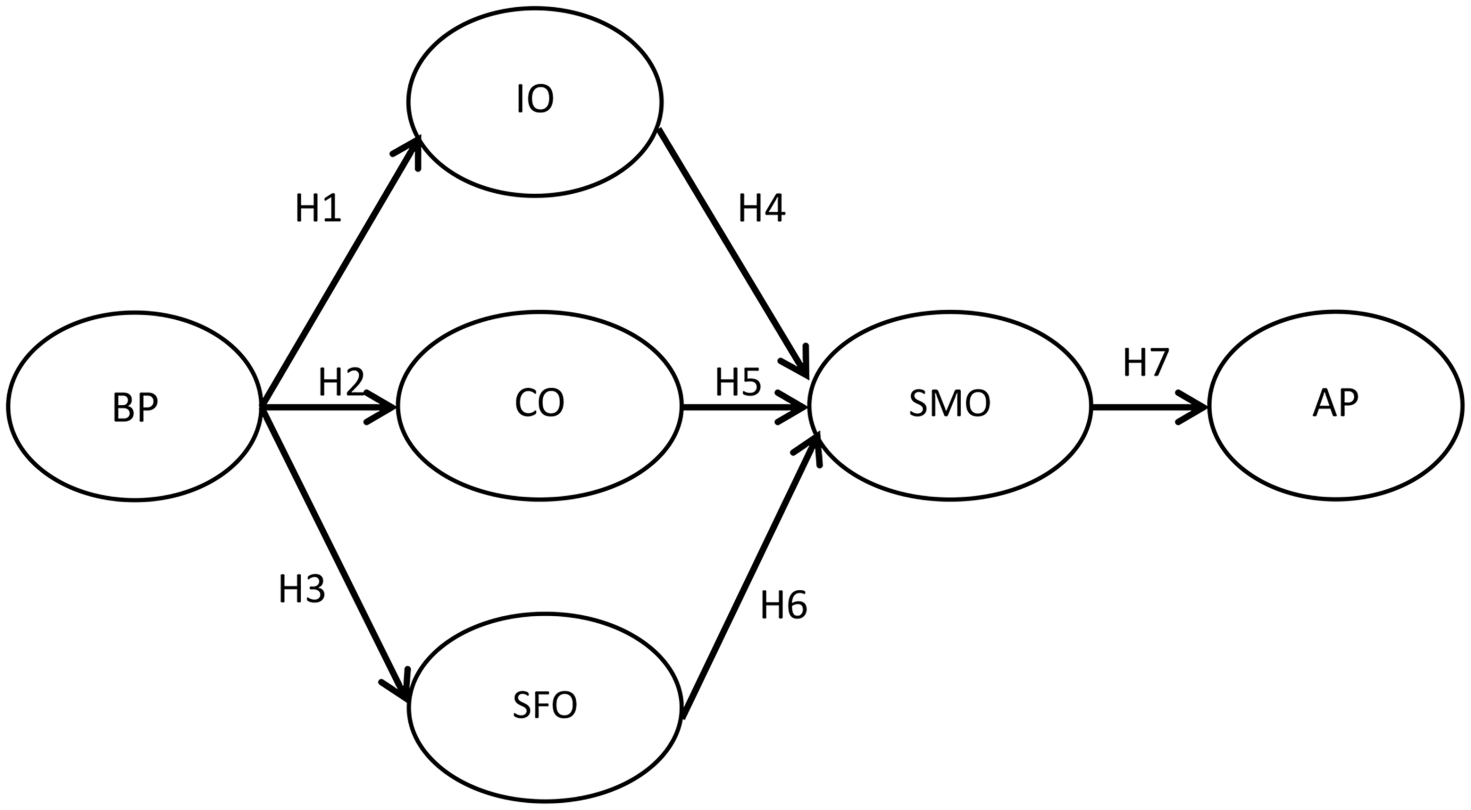
Research framework.

According to the above hypotheses, the research framework is shown in Figure 1.

## Data collection

We invited college students to engage in the survey through publishing questionnaires on i.chaoxing.com to collect data online. Every participant was informed that their answers would be kept confidential. Thanks to the suspension of offline courses during the lockdown of the city, data were collected for 2 weeks in September 2021. In order to enhance the sample representativeness, researchers select effective sample clusters based on their research purposes and issues. Thus, purposive sampling is adopted, and several conditions will be established during sampling so as to improve the representativeness of the research samples. First, mainland China, where the pandemic was most severe in the beginning, was selected as the main area for sampling, and the quarantine policy was the strictest. Thus, it is representative to a certain extent. Second, to understand the psychological characteristics of college students, it is necessary to focus on those who actually face boredom proneness. Third, while filling the questionnaire, all the samples were already isolated at home. In order to attract broader participation of college students, we offered a coupon valuing two dollars for each participant who completes the questionnaire. Students were required to provide their student number to avoid the repeat submission. All variables were adopted from prior literature and were measured on a five-point Likert scale with response choices ranging from “Strongly disagree (1)” to “Strongly agree (5)” In order to screen social media users from participants, we design a question: Are you a user of some social media? Only the questionnaires submitted by those who answered yes to this question are considered as the valid ones. A total of 500 students participated in this survey, and 15 of them were excluded from the sample pool because they do not use social media. Besides, additional 44 questionnaires were not completed. As a result, we finally obtained 441 valid samples.

When self-report questionnaires are used to collect data at the same time from the same participants, common method variance (CMV) may be a concern. A *post hoc* Harman one-factor analysis was used to test for CMV (Podsakoff and Organ, 1986). The explained variance in one factor is 40.554%, which is smaller than the recommended threshold of 50%. Therefore, CMV is not problematic in this study to test the research model, a survey instrument was developed with each construct measured using multiple items (Harman, 1976). Most items were adapted from existing measures in the related literature with confirmed content validity and reliability, and then modified to fit our research context. Boredom proneness was measured by four items adapted from Vodanovich et al. (2005). Information overload were measured by four items adapted from Karr-Wisniewski and Lu (2010). Communication overload were measured by four items adapted from Karr-Wisniewski and Lu (2010). System feature overload was measured by three items adapted from (Lee et al., 2016). Social media overload was measured using Maier et al. (2015) instrument (four items). Academic performance uses the scales (four items) developed by Welbourne et al. (1998). All items were measured with a five-point Likert scale (1, totally disagree; 5, totally agree).

### Evaluation of measurement model

Data analysis was divided into two stages: the reliability and validity of the measurement model were evaluated in the first stage, and the structural model was examined in the second stage to conduct the examination of the research hypotheses (Hair et al., 2009). In this study, the latent variable structural equation models (SMEs) of SmartPLS3.0 and SPSS 25 were adopted as the analysis method. Currently, academics generally agree with the approach of Anderson and Gerbing (1984). That is, CFA should report Standardized Factor Loading, Multivariate Correlation Squared, Composite Reliability, and Average Variance Extracted for all variables, and only after these metrics pass the test can structural models be evaluated. Currently, academics generally agree with the approach of Anderson and Gerbing (1984). That is, CFA should report Standardized Factor Loading, Multivariate Correlation Squared, Composite Reliability, and Average Variance Extracted for all variables, and only after these metrics pass the test can structural models be evaluated. Table 1 shows the average number, factor loading, reliability, and average variance extracted (AVE) value of each construct in this study. The composite reliability (CR) of each construct in this study ranged from 0.870 to 0.954, and every Cronbach’s alpha (α) was higher than 0.7, indicating a high reliability for the constructs in this study. AVE ranged from 0.791 to 0.897, which was also greater than 0.500, indicating a good convergent validity for the constructs in this study. Table 2 shows the correlation coefficient matrix for each construct in this study. The square root of the AVE for each construct was greater than the correlation coefficient for the dimensions (Chin, 1998), indicating a good discriminant validity for the constructs in this study. Besides, Henseler et al. (2016) have proposed that the heterotrait-monotrait ratio (HTMT) of correlations based on the multitrait-multimethod matrix could be adopted as a method to determine discriminant validity. Table 3 shows that the HTMT values for the constructs are all lower than 0.9, showing a good discriminant validity for the constructs in this study. The above analysis showed a good construct validity for this study.

**Table 1:** Summary of study measures and factor loadings of CFA and SEM.

Constructs	Standardized loadings	*α*	CR	AVE
Boredom proneness (BP)		0.851	0.870	0.688
BP 1	0.839			
BP 2	0.739			
BP 3	0.869			
BP4	0.865			
Information overload (IO)		0.877	0.913	0.725
IO 1	0.848			
IO 2	0.888			
IO 3	0.848			
IO4	0.819			
Communication overload (CO)		0.876	0.891	0.729
CO 1	0.854			
CO 2	0.871			
CO 3	0.865			
CO 4	0.826			
System feature overload (SFO)		0.789	0.940	0.703
SFO 1	0.822			
SFO 2	0.832			
SFO 3	0.860			
Social media overload (SMO)		0.859	0.954	0.789
SMO 1	0.837			
SMO 2	0.861			
SMO 3	0.826			
SMO 4	0.828			
Academic performance (AP)		0.911	0.953	0.870
AP 1	0.849			
AP 2	0.920			
AP 3	0.879			
AP 4	0.902			

SD, standard deviation; CR, composite reliability; AVE, average variance explained.

**Table 2:** Matrix of construct correlation coefficients.

Constructs	1	2	3	4	5	6
1. IO	0.851					
2. AP	−0.083	0.888				
3. BP	0.465	−0.146	0.829			
4. CO	0.792	−0.088	0.525	0.854		
5. SMO	0.523	−0.239	0.560	0.536	0.838	
6. SFO	0.798	0.015	0.392	0.686	0.442	0.838

The diagonal line is the square root of the average variance extracted (AVE) of each dimension.

**Table 3:** Heterotrait-monotrait (HTMT).

Constructs	1	2	3	4	5	6
1. IO						
2. AP	0.090					
3. BP	0.521	0.162				
4. CO	0.802	0.098	0.588			
5. SMO	0.601	0.260	0.635	0.616		
6. SFO	0.859	0.031	0.455	0.820	0.532	

### Data analysis and results

Smart PLS 3.0 was adopted for the structural model analysis in this study. The value of the standardized root mean square residual (SRMR) can be applied to evaluate the fit of the research model, which is between 0 and 1: the closer it is to 0, the better the fit is. However, the saturated model of the SRMR assumes that the number of paths in the structural model is the same as the number of related constructs in the measurement model, and the estimated model is calculated in terms of the sample dataset itself and the rows. When the SRMR of the saturated model and the estimated model is less than 0.08, it indicates a good fit for the model Hu and Bentler (1999). The value of the normed fit index (NFI) is between 0 and 1, where the closer it is to 1, the better the fit is, and a value of NFI greater than 0.8 means an acceptable fit.

According to the results calculated by Smart PLS, the value of the SRMR for the saturated model in this study is 0.056, and the value of the SRMR for the estimated model is 0.064, both of which are less than 0.080. The value of NFI is 0.842, which meets the requirements for fit. There is thus a good model fit for this study.

**Figure 2:**
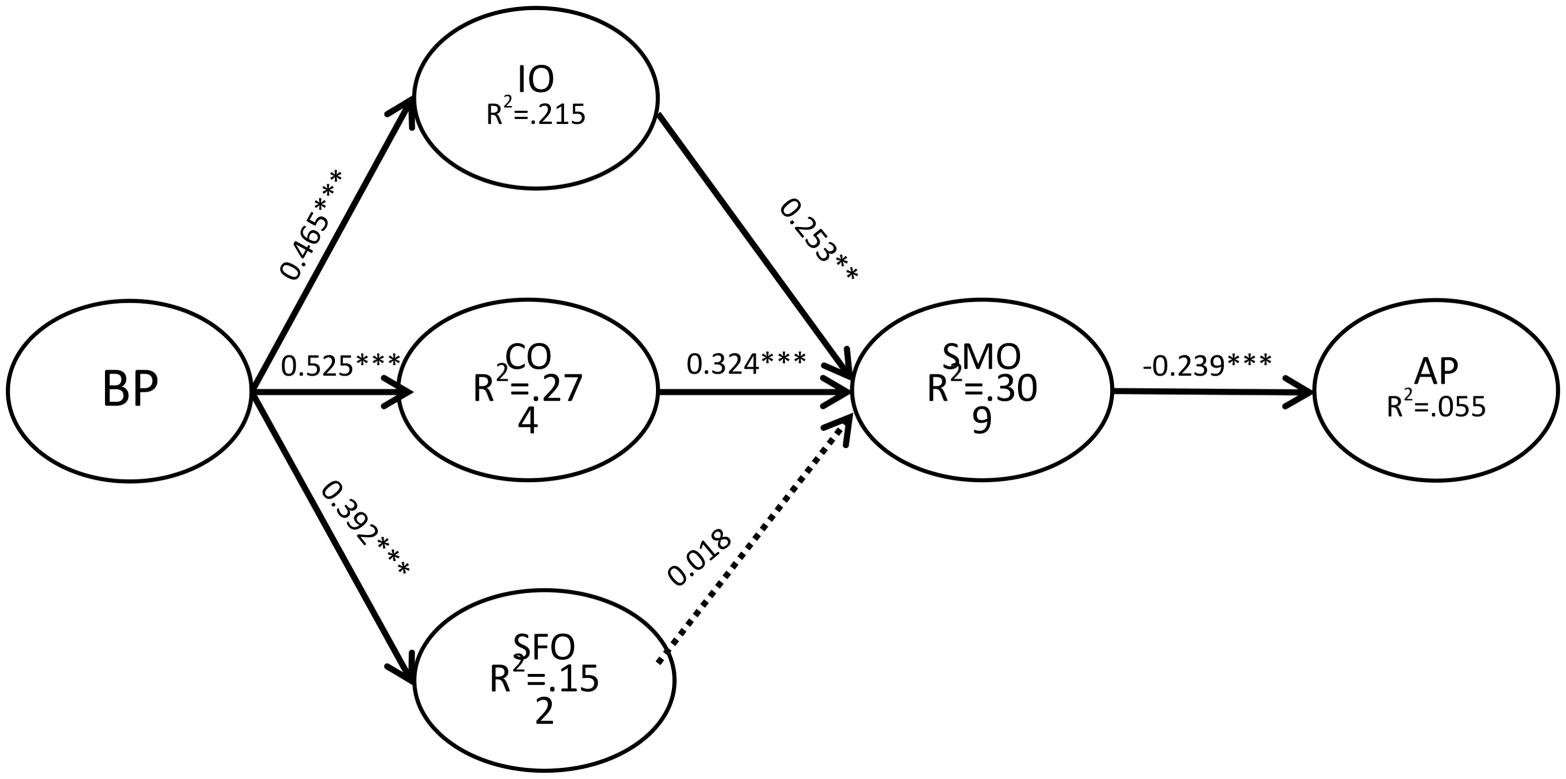
SEM analysis of the research model.

After the evaluation and measurement results were found to be satisfactory, we evaluated the structural model, and examined the hypothesis through the percentage of variance and the significance of structural path. Figure 2 shows the test results of the PLS analysis containing control variables. Boredom Proneness was positively correlated with information overload (*β* = 0.456, *p* < 0.001), communication overload (*β* = 0.525, *p* < 0.001) and system feature overload (*β* = 0.392, *p* < 0.001), thus supporting H1, H2, and H3. Information overload (*β* = 0.253, *p* < 0.01) and communication overload (*β* = 0.324, *p* < 0.001) had a significant positive correlation with the sense of Social Media Overload, thus supporting H4 and 5. Social media overload (*β* = −0.239, *p* < 0.001) was positively correlated with independent academic performance, thus supporting H7. But system feature overload (*β* = 0.018, *p* = 0.124) is not significant for social media overload, so H6 is not supported.

## Discussion

This study reveals that boredom proneness is correlated with social media overload can lead to reduced academic performance among college students. Our analysis of data collected from 441 college students confirms that boredom proneness is positively correlated with information, communication and system feature overloads as well as social media overload and it weaken students’ academic performance.

College students tend to be negatively susceptible to social media because of their flexible timetable, lots of free time and lack of self-control, especially when bored during the quarantine and lockdown period. Recent studies have observed the harmful effects of social media overload (Lutz et al., 2014; Bright et al., 2015; Maier et al., 2015; Dhir et al., 2018), but none of them have given a theoretical explanation on how and why the social media overload leads to reduced performance. This study aims to discuss the impact of social media overload on college students’ academic performance through combining TCIU with the SSO model.

### Theoretical significance

As an expansion of prior studies on the SSO model, this study finds that the boredom proneness which appeared among college students during COVID-19 interacted with its resulting social media overload, which further has caused a decline in students’ academic performance. This study makes multiple theoretical contributions. First of all, we combine TCIU with the SSO model to theoretically understand antecedents and outcomes of social media overload. Our study confirms that the use of the SSO model allows us to better understand the validity of digital technologies and education phenomena. Although prior literature has investigated social media overload through the SSO model and stress–strain–outcome model (Luqman et al., 2017; Loh et al., 2021), we maintain that the SSO model can better illustrate this complex human behavior.

Second, our research findings show that boredom proneness is positively correlated with overload. In terms of practical implications, the findings from this study can be used as justification for developing targeted interventions that enhance cognitive control abilities so as to avoid social media overload and ultimately impaired performance. Current studies of antecedents of social media overload either place emphasis on demographics and usage characteristics (Laumer et al., 2014) or underline system and information characteristics (Cho et al., 2011; Lee et al., 2016). On the contrary, this study verifies the effectiveness of implicit stimuli in explaining social media overload, which means that boredom proneness may finally cause the social media overload that adversely affects college students’ academic performance. Our results show that information overload and communication overload significantly herald social media overload. In essence, social media overload is a transient phenomenon, and it takes a period of time for users to reach the overload state. The inability to control social media use and override impulsive judgments will finally lead to overload. Thus, we conclude that the problematic use of technologies will result in an imbalance between cognitive processes (Soror et al., 2015; Turel et al., 2016). This study also finds that social media overload has a negative impact on academic performance. Although most of the prior literature uses social media overload as a mechanism to explain problematic social media use (LaRose et al., 2003, 2014), our study proves that social media overload will eventually lead to a decline in students’ academic performance, which is an extension of current knowledge.

However, system feature overload has no effect on social media overload, which is contrary to our expectations. Some scholars have indicated that individuals are more willing to use diversified system features to form a relatively special work style and are more tolerant of the corresponding increase in cognitive load and interference caused by system features. In addition, some prior studies also reveal that individuals may be accustomed to continuous and high system function overload after forming an intimate relationship with their mobile devices in life (Yoo, 2010; Bødker et al., 2014). As argued in previous studies, system feature overload will reduce as the age lowers (Zhang et al., 2016). A possible explanation is that college students are more interested in social network services, which makes them not susceptible to system feature overload.

Third, by discussing the dark side of social media use among college students, this study has enriched the research on cyber psychology and is beneficial for the development of research on technology stress. In the context of the COVID-19 quarantine, studies on social media overload among college students are urgently needed and are important to offer new insights. College students are a major user group of social media, and the focus of relevant studies is placed on how this group actively engages in social media but seldom on how they wrongly use social media (Priporas et al., 2017; Throuvala et al., 2019). Our findings dispel the stereotype that college students can completely take control of social media use on their own, and it foregrounds this generation’s vulnerability in terms of social media use, especially during the pandemic (Turner, 2015). As stated by TCIU, social media have been major recreational tools for college students during the COVID-19 quarantine period. This study attests that overload will result in social media overload from the perspective of users and underlines the potential defects of using social media in college students’ learning processes.

### Practical significance

To achieve our goal, we followed the stress-based behavioral theory proposed in the SSO model (Davis and Luthans, 1980). In this process, this study facilitates research on general explanations for the relationship between social media and college students’ academic performance and gives more detailed and specific explanations for the causality. As expected, boredom proneness results in the overload.

This study has the following practical significances. First of all, parents, educators and the public should raise their awareness of the dark side of social media, especially boredom proneness. Parents and educators need to encourage young social media users (e.g., college students and teenagers) to bravely confront their boredom proneness, motivate them to avoid or get rid of negative boredom and support the positive use of social media as motivation.

Second, our study also offers valuable insights for developers of social media systems. It is well-known that social media developers build in features to distract users from alternate tasks in order to increase fixation with their own service. We found that these overloads lead to social media overload among students, and ultimately negatively affects their academic performance. Other studies report overloaded users are more likely stop using the service altogether. Thus, it makes business sense for social media providers to incorporate features in their service which assist the user in avoiding overload.

Third, although this study does not support the influence of system feature overload on social media overload, we still recommend that social media providers facilitate a smooth transition by announcing the changed schedule and scope in advance to reduce discomfort. Besides, social media providers can also provide user guides to introduce new features to users. It is even suggested that SNS providers allow users to design their own SNS accounts instead of developing complex accounts and then integrating embedded features into these accounts.

### Research limitations and research prospects

There are some limitations to this study. First of all, data were collected at four universities in a particular country, i.e., China. The research results may be different if other age groups, cultural backgrounds and pandemic quarantine stages are considered. To eliminate this limitation, we suggest that other scholars include a similar research model, other age groups with different cultural backgrounds and all the stages of the pandemic in their studies. Second, this is a cross-sectional study that collects data from online groups, so there is the issue of method bias (e.g., selection and response bias), and possible changes to given relationships within a period of time cannot be reflected. To address this limitation and considering the continuity of the pandemic, we suggest using a longitudinal method and/or qualitative method to further explore psychological health and the effects of social media use. Third, overload is considered to be a harmful psychological state in this study, and social media overload is considered as the behavioral outcome of the information overload on social media. However, previous research on the dark side of social media use reveals that psychological illnesses also include other aspects, such as anxiety, depression and tiredness (Dhir et al., 2018; Evers et al., 2020), as well as sleep problems (Dhir et al., 2019; Malik et al., 2020; Tandon et al., 2020). Consequently, we advocate for other scholars to expand the findings of this study by taking other relevant issues into account. Likewise, scholars can also explore how to improve individuals’ psychological well-being and mental health by making use of social media during the pandemic. Finally, gender has been an important influencing factor for studies of college students behaviors, because male students may have a different grasp of social media overload from female students. As a result, this study suggests to consider comparisons between males and females to offer richer and more valuable significance to the development of theoretical models.

## Data availability statement

The raw data supporting the conclusions of this article will be made available by the authors, without undue reservation.

## Ethics statement

The studies involving human participants were reviewed and approved by Academic Committee of Fujian University of Technology. The patients/participants provided their written informed consent to participate in this study.

## Author contributions

All authors listed have made a substantial, direct, and intellectual contribution to the work and approved it for publication.

## Funding

Fujian Social Science Planning youth project. Research on the development mechanism of agricultural e-commerce industry in Fujian Province from the perspective of big data (FJ2020C062).

## Conflict of interest

The authors declare that the research was conducted in the absence of any commercial or financial relationships that could be construed as a potential conflict of interest.

## Publisher’s note

All claims expressed in this article are solely those of the authors and do not necessarily represent those of their affiliated organizations, or those of the publisher, the editors and the reviewers. Any product that may be evaluated in this article, or claim that may be made by its manufacturer, is not guaranteed or endorsed by the publisher.
